# The Bacillary Postbiotics, Including 2-Undecanone, Suppress the Virulence of Pathogenic Microorganisms

**DOI:** 10.3390/pharmaceutics14050962

**Published:** 2022-04-29

**Authors:** Satish Kumar Rajasekharan, Moshe Shemesh

**Affiliations:** Department of Food Science, Institute of Postharvest Technology and Food Sciences, Agricultural Research Organization (ARO), The Volcani Institute, Rishon LeZion 7528809, Israel; generic.sat@gmail.com

**Keywords:** *L. plantarum*, postbiotics, ketones, *C. albicans*, pathogenic hyphae, pathogenic biofilm

## Abstract

Secreted molecules from probiotic *Bacilli* have often been considered potential pharmaceuticals to fight infections caused by bacterial or yeast pathogens. In the present study, we investigated the antagonistic potential of secreted probiotic filtrates (hereafter, postbiotics) derived from *Lactobacillus plantarum* cells against pathogenic microorganisms, such as *Escherichia coli*, *Staphylococcus aureus*, and *Candida albicans*. We found that the postbiotics mitigate the biofilms of the tested pathogens with no notable effect on their planktonic growth. In addition, the postbiotics suppressed some virulence traits, for instance, the dendrite swarming motility of *E. coli* and yeast-to-hyphal switch in *C. albicans*. Further assays with an active constituent produced by the *L. plantarum* cells–2-undecanone revealed two significant findings: (i) 2-undecanone inhibits *C. albicans* biofilms and hyphae *in vitro* and in a *Caenorhabditis elegans* model, and (ii) it interacts specifically with Gln 58 amino acid residue of hyphal wall protein-1 (Hwp-1) in molecular docking analysis. The results suggest the targeted mode of antagonistic action of 2-undecanone against *C. albicans* biofilm. In total, the findings of the study depict an appealing strategy to use postbiotics, including specific ketone molecules, produced by *L. plantarum* for developing novel antibiofilm and anti-hyphal pharmaceuticals.

## 1. Introduction

Biofilms are well-structured, nourished, and self-protected multicellular communities of sessile microbes embedded in an extracellular polymeric matrix [1]. Biofilms can be harmful or beneficial depending on the causative organism [2]. Biofilm-associated infections caused by bacterial or yeast pathogens have been frequent and recurrent threats to humans [3,4]. In several microbial pathogens, biofilm formation is a crucial defense mechanism that often facilitates virulence genes expression and may promote developing resistance to antimicrobial chemotherapy. Therefore, the biofilm mode of growth displays recalcitrant behavior, and exhibits increased tolerance to antibiotics [5], which often leads to the failure of antibiotic therapy and the evolution of multi-drug resisting microbial variants.

Current thinking in the field of biofilm research dictates of need for developing novel approaches to mitigating biofilm formation [6]. Innovative treatment involves using small-molecule inhibitors that either block microbial adhesion, prevent inter- or intracellular microbial communications, or disrupt pre-formed biofilms [7,8]. Lately, such small-molecule inhibitors are being increasingly viewed as potential antibiofilm pharmaceuticals. Thus, studies to identify or/and develop new antibiofilm pharmaceuticals are rapidly intensifying. Another streamlined approach to treating pathogenic biofilms is probiotic therapy [9]. Probiotics are live microorganisms that are supposed to be beneficial in enhancing human welfare [10]. Lactic acid bacteria (LAB) are the most popular probiotics shown to eradicate pathogens by the competitive-exclusion principle [11], i.e., LAB may compete with and successfully eliminate the pathogenic species. For example, in the human vaginal environment, LAB are natural competitors of the yeast pathogen *Candida albicans* [12]. Aided by the low pH of the vaginal environment, LAB may robustly suppress *C. albicans* infection. LAB can also secrete a myriad of volatile compounds or secondary metabolites that may assist in their antagonistic activities against pathogens [13,14].

*Lactobacillus plantarum* is a prospective probiotic bacterium with a Qualified Presumption of Safety (QPS) and a Generally Recognized as Safe (GRAS) status [15]. Thus, *L. plantarum* has often been used as bio-control measures against foodborne pathogens [16]. In addition, cell-free supernatant of *L. plantarum* cultures has been shown to inhibit the growth of *Listeria monocytogenes*, *Salmonella enteritidis*, *Escherichia coli* O157:H7, and *Staphylococcus aureus* [17,18]. Secondary metabolites such as organic acids, bacteriocins, and other antimicrobial peptides secreted by *L. plantarum* in its culture filtrates may account for the observed antimicrobial effects [19,20,21]. Recently, a probiotic *L. plantarum* MG98 strain was shown to inhibit *E. coli* or *C. albicans* during coculture assays [17,22]. This strain also prevented yeast adhesion and colonization on HT-29 cells, thus explaining the importance of *L. plantarum* as a biocontrol against *Candida* infections.

In addition, postbiotics and metabolic byproducts of probiotics from *Lactobacillus* species were shown to reduce biofilm formation by several pathogens, including *Aggregatibacter actinomycetemcomitans*, *Pseudomonas aeruginosa*, and *Listeria monocytogenes* [23,24,25,26,27]. In the current study, we aimed to characterize the antagonistic activities of postbiotics from *L. plantarum* colonies against microbial pathogens, such as *E. coli*, *S. aureus*, and *C. albicans*. Our findings indicate the promising role of the postbiotics prepared from *Lactobacillus plantarum* colonies in the development of antibiofilm pharmaceuticals. Furthermore, the findings also provide *in vitro*, *in vivo*, and *in silico* proof-of-concept for applying bacillary postbiotics, including one of its active compounds, 2-undecanone, as a potent antibiofilm and anti-hyphal agent.

## 2. Materials and Methods

### 2.1. Microbial Strains and Culture Conditions

Microbial strains used in the study are listed in Table S1. *L. plantarum* was maintained and experimented in De Man, Rogosa, and Sharpe (MRS) hard agar (2%) (HiMedia, Maharashtra, India) or liquid media (incubation at 37 °C without shaking). *E. coli* and *S. aureus* cells were grown in Lysogeny broth (LB) (incubation at 37 °C with shaking at 150 rpm), or LB supplemented with 2% agar. *C. albicans* was maintained in potato dextrose agar (PDA) or potato dextrose broth (PDB) and experimented in either PDA/PDB or Roswell Park Memorial Institute medium-1640 (RPMI) (incubation at 37 °C with shaking at 150 rpm).

### 2.2. Preparation of Colony Filtrates and Pure Ketones

Probiotic filtrates (postbiotics, as it was defined above) were prepared from *L. plantarum* colonies grown on acidified MRS for 5–7 days at 37 °C [28]. Briefly, *L. plantarum* colonies (approximately 10 similar-sized colonies of 0.4 cm size) were lifted wholly and transferred to 1 mL sterile distilled water at room temperature. The colonies were vortexed for 15 min and sonicated (2 min (10 s pulse on/off) at 4 °C with 40% amplitude), following which the cells were centrifuged and pelleted at 10,000 rpm for 2 min. The harvested supernatant was filter-sterilized using a 0.2 µm filter and designated as ambient postbiotics (AP). For cold-stressed postbiotics (CSP) preparation, the above-mentioned MRS plates containing colonies were incubated at −17 °C for 1 h, following which a similar procedure was followed as for the AP preparation. Pure ketones (2-undecanone, 2-nonanone, and 2-heptanone) were purchased from Sigma Aldrich (St. Louis, MO, USA).

### 2.3. Biofilm Inhibition Assay

Biofilm inhibition assays were conducted in 96-well polystyrene microtiter plates (Tarsons Products Pvt. Ltd., Kolkata, India) as previously described [29]. Briefly, bacterial or yeast cells were inoculated overnight in either LB or PDB (with 0.05% glucose) at initial turbidity of 0.1 at 600 nm (OD_600_) with or without postbiotics (5% *v*/*v*) and incubated at 37 °C for 24 h. After incubation, the planktonic cells were removed, the biofilm cells (attached to the bottom of polystyrene surface) were washed with distilled water and stained with crystal violet (0.1%) for 20 min. Afterwards, the plates were washed repeatedly, and the residual biofilm cells (attached to the polystyrene surfaces) were dissolved in 95% ethanol. The planktonic growth was measured at 600 nm, while the biofilms were measured at 575 nm using a Biowave CO8000 cell density meter. For microscopic imaging of biofilms attached to the polystyrene surfaces, the wells were stained with SYTO^®^ 9 dye as per manufacturer’s instructions (Filmtracer™ LIVE/DEAD™ Biofilm Viability Kit, Thermo Fisher Scientific, Waltham, MA, USA), and the plates were incubated for 30 min at 37 °C in dark. Following incubation, the cell attached to surfaces were washed with distilled water to extract the residual stain, dried, and surface of the microtiter plates were imaged under Nikon fluorescent microscope (Nikon Eclipse Ti2, Tokyo, Japan) using a GFP (488 nm) emission filter. For biofilm disruption assay, overnight cultures in either LB or PDB were used to generate mature biofilms. After 24 h of incubation, the spent medium was removed, fresh media containing the postbiotics were replaced, and the plates were further incubated at 37 °C for 24 h. Biofilm disruption was monitored using microscopy and CV quantification as described above.

### 2.4. Swarming Motility Assay

Swarming motility was assessed by spotting 2 µL of the overnight culture (*E. coli*) diluted at initial turbidity of 0.1 at 600 nm (OD_600_), onto the center of the Petri plates containing semi-solid motility agar medium (1% tryptone, 0.25% NaCl, and 0.5% agar) supplemented with or without AP or CSP (5% or 10% *v*/*v*). The plates were incubated for 37 °C for 24 h, while the subsequent branching pattern (in non-treated groups) was measured, compared (with treatment groups), and photographed using a Huawei p30 pro smartphone (Huawei VOG-L29 camera).

### 2.5. Yeast–Hyphae Switching Assay

Yeast–hyphae (Y–H) switching assays were conducted in liquid RPMI-1640 medium as previously described [29]. Briefly, overnights cultures of *C. albicans* (grown in 2 mL of PDB) were inoculated in RPMI-1640 at initial turbidity of 0.1 at 600 nm (OD_600_) and treated with or without postbiotics (5 and 10% *v*/*v*) or ketones (0.005–0.1%). The cultures were then incubated at 37 °C with shaking (150 rpm) for 24 h. Following incubation, 5 µL of the cultures were transferred to the microscopic slide and imaged under Nikon fluorescent microscope (Nikon Eclipse Ti2, Japan) using differential interference contrast (DIC) filter.

### 2.6. Assay of Colony Morphology in Hard Agar

Colony morphology was analyzed by streaking *C. albicans* on PDA agar plates containing postbiotics (5 and 10% *v*/*v*) or ketones (0.005–0.1%), in comparison to the not-treated groups. The plates were incubated at 37 °C for 7 days, and the hyphal prostration from colony edges was assessed using a phase-contrast mode of the Nikon fluorescent microscope (Nikon Eclipse Ti2, Japan).

### 2.7. Caenorhabditis elegans Toxicity Assay

*Caenorhabditis elegans* wild-type strains were maintained on nematode growth medium (NGM) with *E. coli* OP50 as a regular feed. In order to assess the toxicity of postbiotics (10% *v*/*v*) or 2-undecanone (0.01%), synchronized adult nematodes were reared and tested in a 96-well microliter plate as previously described [30]. The control and treatment groups (each consisting of approximately 30 nematodes per well) were suspended in liquid M9 buffer and monitored for 7 days. The live or dead nematodes were counted using the Nikon fluorescent microscope (Nikon Eclipse Ti2, Japan) using a DIC and DAPi (blue LED light) filters, and the survival percentage was calculated.

### 2.8. Candida albicans-Caenorhabditis elegans Infection Model

An *in vivo* infection model was used for assessing the role of 2-undecanone in restraining *C. albicans* infections in *C. elegans* as previously described [30]. In brief, overnight cultures of *C. albicans* were used to prepare lawns on potato dextrose agar (PDA) plates and incubated for 48 h at 37 °C. Synchronized nematodes were allowed to feed on *C. albicans* lawn for four hours at 25 °C, after which time the worms were collected and washed with M9 buffer. In 96-well microtiter plates containing 200 mL of PDB media, approximately 30 nematodes were pipetted into each well and treated with 2-undecanone at a concentration of 0.01%. Controls were maintained in the PDB medium alone. The survival rates of the nematodes were monitored for 7 days, and the results were expressed as percentages of live nematodes following three independent measurements.

### 2.9. Molecular Docking Assay

Computational studies were performed to elucidate the interactions of methyl-3-ketones (2-undecanone, 2-nonanone, and 2-heptanone) and standard hyphal inhibitors with an Hwp1 (hyphal wall protein 1) as previously described [31] with a slight modification. The protein sequence was retrieved from UniProt (P46593) and I-TASSER online server used for the protein modeling from multiple threading alignments. The molecular docking was performed using Schrödinger Maestro 11.4 (Schrodinger Software Solutions, New York, NY, USA). The procedure involved the following steps: (a) ligprep, (b) protein preparation wizard, (c) glide grid generation, and (d) docking. The grid was generated in close proximity to the active sites, and docking was executed using the Glide (grid-based ligand docking energetic) module. Interactions were visualized using Incentive PyMOL viewer (v1.8.2.3).

### 2.10. Gas Chromatography/Mass Spectrometry (GS-MS) Analysis

To further determine antagonistic potential of *L. plantarum* colonies, they were grown on MRS agar in special glass jars with elastic septum seal at their top. A glass jar with sterile MRS served as control. After incubation at 37 °C for 24, 48, or 72 h, SPME fiber (solid-phase microextraction (SPME) using stable-flex fibers, 1 cm in length, coated with a 50/30-μm layer of divinylbenzene/carboxen/polydimethylsiloxane (DVB/CAR/PDMS) (Supelco, Bellefonte, PA, USA)) was inserted into the jar, and this complex was incubated in 50 °C for 30 min in order to allow the adsorption of the volatiles into the fiber. After incubation, manual injection took place, and the fiber was desorbed for 1 min at 250 °C in the splitless inlet of the GC (Agilent 7890A, Palo Alto, CA, USA) equipped with an HP-5 column (30 m × 0.25 mm ID, 0.25 μm film thickness, J&W Scientific, Folsom, CA, USA). The oven was programmed for initial temperature of 40 °C for 1 min, and then ramped to 150 °C at 5 °C min^−1^, and to 270 °C at 15 °C min^−1^ and held at that temperature for 1.3 min. The helium carrier gas flow was set at 0.8 mL min^−1^. The effluent was transferred to an MS detector (Agilent 5975C, Palo Alto, CA, USA) that was set to scan from mass 40 through 300 at 7.72 scans/s in the positive-ion mode, and mass spectra in the electron impact (EI) mode were generated at 70 eV. Chromatographic peaks were identified by comparing the mass spectrum of each component with the US National Institute of Standards and Technology (NIST) library of mass spectra, 2006 version. Volatile’s composition was similar in samples incubated for 7 days.

### 2.11. Statistical Analysis

All experiments were performed in triplicates, and results are expressed as means ± standard deviations. The Student’s *t*-test was used to determine the significance of differences between treated and non-treated groups. Statistical significance was accepted for *p* values < 0.05, and significant changes are indicated using asterisks in figures (* *p* < 0.05, ** *p* < 0.01, and *** *p* < 0.001).

## 3. Results

### 3.1. Bacillary Postbiotics Restrain Biofilm Formation by the Enteropathogenic Bacteria

**Figure 1:**
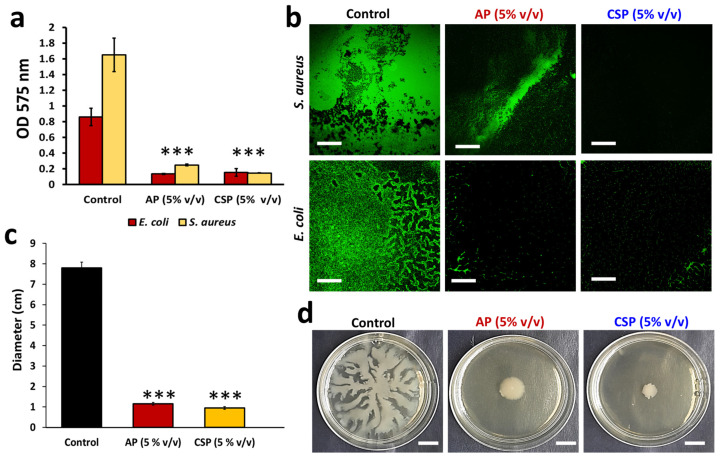
Effect of Bacillary postbiotics on physiology of bacterial pathogens. (**a**) Effect of AP and CSP on *E. coli* and *S. aureus* biofilm formation. Biofilms were generated on polystyrene surfaces in the presence or absence of the postbiotics, visualized, and quantified by crystal violet staining method. The graph shows the means ± SEMs of three measurements. *** *p* < 0.001 vs. the non-treated controls. (**b**) Fluorescent microscopic images of bacterial biofilms on polystyrene surfaces treated with either AP or CSP (5% *v*/*v*). Scale bar: 200 µm. (**c**) The diameter of *E. coli*. swarm area in AP or CSP treated and non-treated controls. The graph shows the means ± SEMs of three measurements. *** *p* < 0.001 vs. the non-treated controls. (**d**) Effect of AP or CSP (5% *v*/*v*) on dendrite swarming motility of *E. coli*. Scale bar: 2 cm.

We initiated this investigation with a hypothesis that certain constituents of cell-free supernatants derived from the probiotic *L. plantarum* could provide promising antagonistic activities against pathogenic microorganisms. First, we assessed the effect of differentially collected postbiotics (AP or CSP, as described in Section 2.2) against two bacterial pathogens, namely *E. coli* and *S. aureus*. The tested postbiotics did not show any significant bactericidal effect on the tested pathogens at the tested dosage (Figure S1). However, we noted a drastic decline in the biofilm-forming ability of the pathogen when grown in the presence of either AP or CSP (Figure 1a). Both postbiotics (AP or CSP) showed a similar effect, indicating that cold stress does not improve notably the antagonistic activities. Further, we confirmed the inhibition of biofilm surface area on a polystyrene substrate by fluorescent microscopic analysis (Figure 1b). The results pinpoint that the tested postbiotics might contain putative signals (metabolites) that disarm biofilms without compromising the planktonic cells. Such molecules could be a prerequisite to the antibiofilm drug discovery.

### 3.2. The Bacillary Postbiotics Inhibit Dendritic Swarming Pattern in E. coli

One of the mechanisms controlling biofilm formation is related to swarming motility regulated by the quorum-sensing (QS) system [31,32,33]. Bacteria may slide on semi-solid agar to generate different types, including a dendritic pattern of swarming motility [33]. The swarming motility is a QS-controlled phenotype that is usually driven by flagellated motion on semi-solid surfaces [34]. We tested the effect of collected postbiotics on the *E. coli* colony expansion characterized by a dendritic pattern of swarming motility (Figure 1c,d). The *E. coli* cells, however, failed to produce the dendrite pattern when AP or CSP was added to the semi-solid agar (Figure 1d). The finding hints at the ability of the postbiotics to restrain swarming motility in *E. coli*. Further work is warranted to understand if the swarming inhibition is related to the QS quenching.

### 3.3. The Postbiotics Mitigate Candida albicans Biofilms

**Figure 2:**
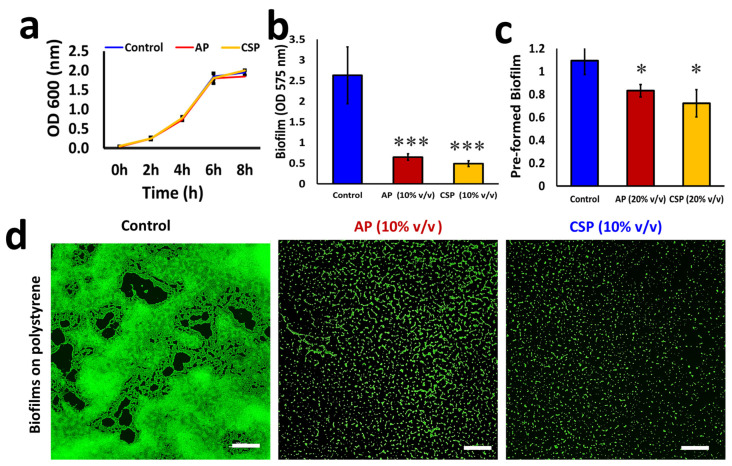
Effect of AP and CSP on growth, biofilms and pre-formed biofilms of *Candida albicans*. (**a**) Growth profile of *C. albicans* in the presence and absence of AP or CSP (10% *v*/*v*). (**b**) Crystal violet quantifications of *C. albicans* biofilm formation on polystyrene surface in the presence and absence of AP or CSP (10% *v*/*v*). The graph shows the means ± SEMs of three measurements. *** *p* < 0.001 vs. the non-treated controls. (**c**) Effect of AP or CSP (20% *v/v*) on pre-formed mature biofilms of *C. albicans.* The graph shows the means ± SEMs of three measurements. * *p* < 0.05 vs. the non-treated controls. (**d**) Fluorescent microscopic images of *C. albicans* biofilms on polystyrene surfaces treated with AP or CSP (10% *v*/*v*). The microtiter plates containing 24 h *C. albicans* biofilms were stained with SYTO™ 9. Scale bar: 100 µm.

*C. albicans* is a remarkable pathogenic yeast model that displays multifarious phenotypes such as biofilm formation, hyphae protruding, filamentation, and flocculation [29]. This organism has often been used as a model for studying the antipathogenic activities of desired chemical, synthetic or natural products. We first sought to assess the biofilm formation ability of *C. albicans* in the presence of collected Bacillary postbiotics. We observed a substantial reduction in the biofilm formation on polystyrene surface in the presence of either AP or CSP, as quantified by crystal violet staining (Figure 2b), whereas the postbiotics did not show a notable fungicidal activity on *C. albicans* (Figure 2a). We further confirmed the biofilm inhibitory activities of AP or CSP against the pathogen using fluorescent staining and microscopic imaging (Figure 2d). Though the postbiotics were effective in preventing the biofilm formation by *C. albicans*, they were not strong enough to disassemble the pre-formed mature biofilm at the tested dosage (10% *v*/*v*). However, a significant reduction (* *p* < 0.05) in the mature (24 h) biofilm was noted when the concentration of postbiotics was doubled (Figure 2c). This indicates the dose-dependent antibiofilm activity of the tested postbiotics.

### 3.4. The Postbiotics Prevent Yeast-to-Hyphal Switching and Hyphal Protrusion from Colony

**Figure 3:**
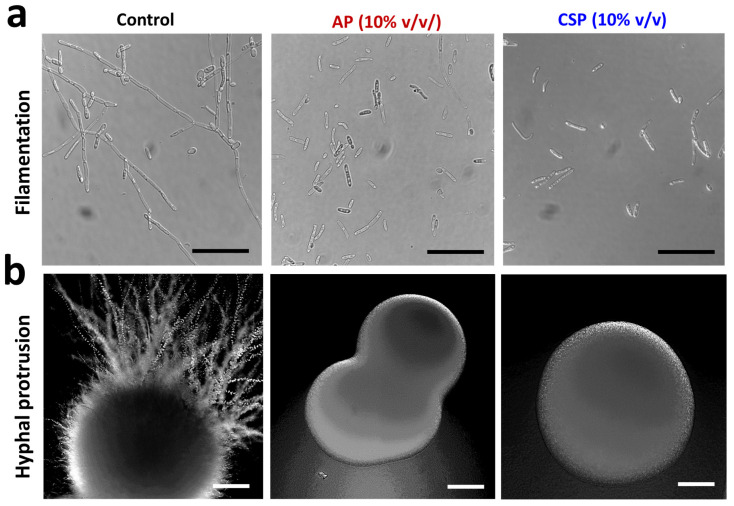
Microscopic images of *Candida albicans* colonies and hyphal filaments. (**a**) Effect of AP or CSP (10% *v*/*v*) on yeast-to-hyphal transition (filamentaion) in *C. albicans* grown in liquid RPMI-1640 media. Scale bar: 20 µm. (**b**) Effect of AP or CSP (10% *v*/*v*) on hyphal protrusion from colony edges of *C. albicans* grown on potato dextrose agar (PDA). The plates containing colonies were incubated for 7 days at 37 °C. Scale bar: 200 µm.

The yeast-to-hyphal (Y–H) switching is dimorphic plasticity displayed by *C. albicans* [29]. The hyphal mode is regarded as a virulence attribute, while the yeast mode is considered commensal [35]. We assessed the dimorphic switching of *C. albicans* in RPMI, a hyphal inducing liquid media in which *C. albicans* grows as an elongated hyphal filament (Figure 3a). In the AP or CSP treated groups, the filamentation was substantially reduced (Figure 3a). We also assessed the hyphal protrusion from colony edges on hard agars. Prolonged incubation (3b) in potato dextrose (PDA) hard agars resulted in hyphal protrusions in control groups, which were absent in treated groups (Figure 3b). The treated groups showed smooth round colony phenotypes indicating that the tested postbiotics are efficient in suppressing filamentation in hard agars as well.

### 3.5. 2-Undecanone, an Active Component Secreted by L. plantarum, Refrains C. albicans Physiology

**Figure 4:**
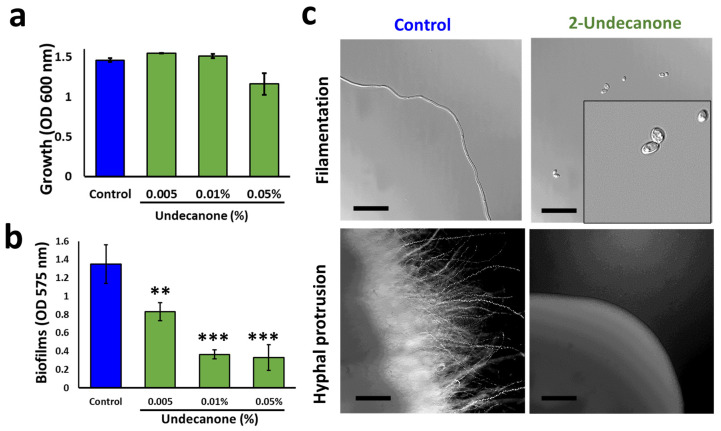
Effect of 2-undecanone on *Candida albicans* physiology. (**a**) Growth profile of *C. albicans* in the presence and absence of 2-undecanone in potato dextrose broth (PDB). (**b**) Crystal violet quantifications of *C. albicans* biofilm formation on polystyrene surface in the presence or absence of 2-undecanone in PDB. The graph shows the means ± SEMs of three measurements. *** *p* < 0.001 and ** *p* < 0.01 vs. the non-treated controls. (**c**) Effect of AP or CSP (10% *v*/*v*) on yeast-to-hyphal transition (filamentations), and hyphal protrusion from colony edges in *C. albicans* grown in liquid RPMI-1640 media and PDA agar, respectively. Scale bar: 200 µm.

*L. plantarum* is known to produce diverse volatile compounds [36], which were shown to possess antibacterial and/or antifungal activities [37]. Our GC-MS analysis revealed the occurrence of several compounds, including methyl-2-ketones derived from the *L. plantarum* colonies, namely: 2-undecanone (Figure S5) [14]. In an effort to identify a specific ketone molecule with antimicrobial activity, we tested the effect of pure ketones (2-undecanone, 2-nonanone, and 2-heptanone) on *C. albicans* physiology. Following ketone molecules, 2-nonanone and 2-heptanopne did not show any notable effect on either biofilm (Figure S2) or hyphal growth (data not shown). Nonetheless, 2-undecanone, even at the lowest dosage (0.005%), was successful in mitigating the biofilm formation of *C. albicans* (Figure 4b) without compromising the planktonic growth (Figure 4a and Figure S3). At higher dosage (0.05 or 0.01%), it showed a substantial decline in the growth profiles as well in RPMI media (Figure S4). Further, 2-undecanone effectively controlled Y–H transitions in liquid RPMI media (Figure 4c). Overall, the biofilm formation, growth, and hyphal morphology were significantly hindered by 2-undecanone in a dose-dependent manner. This confirms that methyl-2-ketones and the Bacillary postbiotics might play a potent role in suppressing *C. albicans* pathogenesis.

### 3.6. The Postbiotics and 2-Undecanone Appear as Non-Toxic in a C. elegans Model

**Figure 5:**
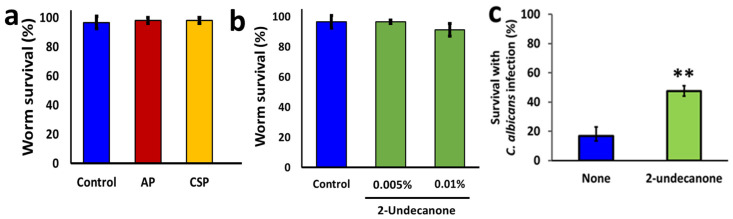
Assessment of toxicity profile of AP, CSP and 2-undeconone in a *Caenorhabditis elegans* model. (**a**) Effect of AP or CSP (10% *v*/*v*) on *C. elegans* survival. The graph shows the means ± SEMs of three measurements. (**b**) Effect of 2-undecanone (10% *v*/*v*) on *C. elegans* survival. The graph shows the means ± SEMs of three measurements. (**c**) Effects of 2-undecanone (0.01%) on survival of *C. elegans* exposed to *C. albicans* for a period of seven days. ** *p* < 0.01 vs. the non-treated controls.

Next, we assessed the toxicity of the Bacillary postbiotics and 2-undecanone on *C. elegans*. Toxicity testing in a biological system is a foremost criterion for pharmaceutics and drug development research. We, therefore, adopted the *C. elegans in vivo* experimental system, which is viewed as a universal model assessing the toxicities and is an effective alternative to rodents [37,38]. Our results suggest that the postbiotics (10% *v*/*v*) and 2-undecanone (0.1%) do not induce any mortality to the tested nematodes (Figure 5), thus regarding them as potentially safe for further investigation in developing antibiofilm technology.

### 3.7. 2-Undecanone Suppresses C. albicans Pathogenicity in a C. elegans Model

We further tested if 2-undecanone could suppress *C. albicans* pathogenicity in a *C. elegans* model by infecting *C. elegans* with *C. albicans*. In this model, infected-*C. elegans* showed only 20% survival (80% fatality) after 7 days of incubation, suggesting that the infection with *C. albicans* is fatal for the nematodes. Interestingly, administration of 2-undecanone (0.01%) enhanced the survivability of infected *C. elegans* to 50% in treated groups (Figure 5c), suggesting that 2-undecanone eliminates *C. albicans* induced killing of nematode, possibility by suppressing the biofilm or hyphal colonization within the nematode.

### 3.8. 2-Undecanone Interacts Profoundly with Hyphal Wall Protein 1 of C. albicans

**Figure 6:**
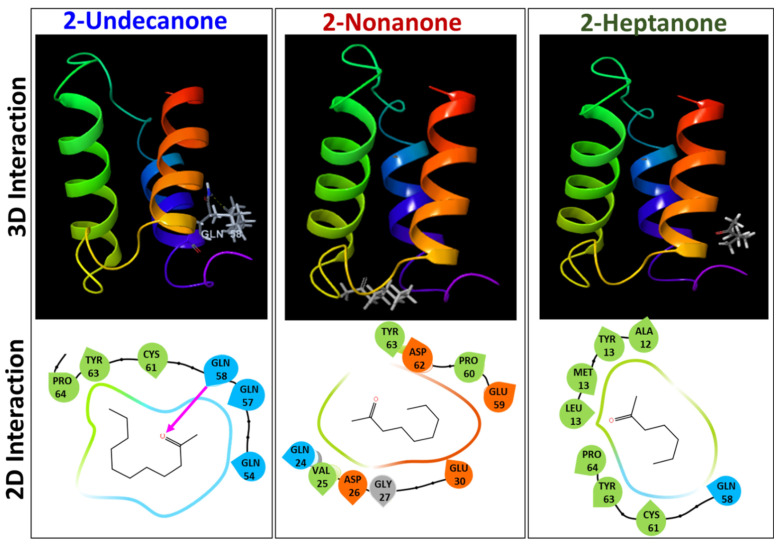
Molecular interaction of ketones with hyphal wall protein 1 (Hwp1 protein). The 3D and 2D interaction diagram showing interactions between ligands (2-undecanone, 2-nonanone, and 2-heptanone) with the active sites of hyphal wall protein 1 (Hwp1). Pink arrow represents the H-bonds.

Finally, we assessed molecular interactions of possible ligands (2-undecanone, 2-nonanone, and 2-heptanone) with the hyphal wall protein 1 (Hwp1), which has been regarded as a major hyphal cell wall protein required for *C. albicans* adhesion, biofilm formation and hyphal development [31,39]. It is suggested that interactions of compounds with the active sites of Hwp1 can lead to protein inactivity. In our model, 2-undecanone was able to interact with Hwp1 by forming a hydrogen bond with Gln 58 amino acid residue in the active site of Hwp1, with a glide score of −4.672 kcal/mol (Figure 6). The surface/volume image is depicted in Figure S6. Other ketones analyzed (2-nonanone (−3.294 kcal/mol) and 2-heptanone (2.943 kcal/mol) did not form any hydrogen bonds and revealed poor binding interactions. The results partially support our *in vitro* observations (antibiofilm and anti-hyphal activities) with the ketones (Figure 4c), thus largely confirming the mode of action of 2-undecanone. Further in-depth analysis is warranted to confirm this observation.

## 4. Discussion

The probiotic *Bacilli,* for instance, *L. plantarum* may produce and secrete a myriad of volatile compounds and secondary metabolites in the vicinity of its existence that often exhibits antibacterial or antifungal activities [36]. The cell-free supernatants from *L. plantarum* have been shown to inhibit several pathogenic microbes [40,41]. Some of them could prevent the formation of pathogen biofilms [40,42]. In this study, we assessed the antagonistic activity of the cell-free postbiotics from *L. plantarum* colonies against bacterial and yeast pathogens. The tested postbiotics did not exert notable bacteriostatic and fungistatic activities, but drastically affected the biofilms of the tested pathogens. Studies have shown that *L. plantarum* LR/14 exhibited profound anti-*Candida* activity [43]. Here, we show for the first time that the postbiotics (intracellular and extracellular secretions) from *L. plantarum* colonies exhibit antibiofilm and anti-hyphal activities without affecting the growth of planktonic counterparts (Figure S1 and Figure 4a). It is conceivable that the concentrations of the metabolites in the cell-free postbiotics are substantially low. That said, our findings demonstrate an antibiofilm effect displayed by the postbiotics, which is a promising alternative for antimicrobial approaches.

Next, we addressed the anti-hyphal activities of the culture filtrates. The Y–H switching is a dimorphic trait by which *C. albicans* helms to manifest its pathogenicity [29]. The hyphal mode represents the aggressive phase of *C. albicans* infection. In humans, hyphae induce tissue damage by an invasion of epithelial cells, causing cytolysis or cell death [44]. In laboratory models such as *C. elegans*, *C. albicans* hyphae pierce through nematode cuticles that progressively kill them [45,46]. *C. albicans* virulence can be alleviated if the hyphae are degraded or neutralized. Several studies are focused on screening chemicals/natural products, synthesizing newer drugs, or repurposing drugs to degrade hyphae or, at a minimum, to prevent Y–H transition [29,47]. The tested postbiotics in this study prevented hyphal filamentations in liquid RPMI and on hard agar, indicating that the postbiotics contain chemicals that function as anti-hyphal agents. Separately, we also identified methyl-2-ketones, 2-undecanone, secreted from 7-day *L. plantarum* colonies by GC-MS profiling (Figure S5). A recent study revealed that methyl-2-ketones were the active volatile chemicals in essential oils (EO) of *Ruta graveolens* and *R. chalepensis* plants that showed profound anti-*C. albicans* activity [48,49]. Furthermore, ketoacids such as nonanoic acid and heptanoic acid were reported to show anti-hyphal and antibiofilm activities against *C. albicans* [50]. The methyl-2-ketones might also account for anti-*Candida* and antibacterial activities. One of the ketones, 2-undecanone, was effective in controlling Y–H transitions and in preventing biofilm formations in *C. albicans*. Studies have shown that blocking Hwp-1 can prevent biofilm formation [20,39]. Recently, the molecular interaction of a natural product, morin, was assessed against the Hwp-1. The study revealed a tight binding interaction of morin with the active site of Hwp-1 facilitated by a hydrogen bonding with Gln amino acid residue [20]. In our model, 2-undecanone interacted with Hwp-1 active sites and formed a hydrogen bonding with Gln 58 amino acid residue. Interestingly, other ketones (2-nonanone and 2-heptanone) did not form any hydrogen bonds, thus confirming the importance of the Gln hydrogen bond in maintaining tight ligand-protein interactions that govern the inactivity of the protein. The docking results rationalize the biofilm inhibitory activity of 2-undecanone and partially confirm that the ketone interacts with Hwp-1, and inactivates its function, consequently preventing biofilm inhibition by *C. albicans*. Consequently, the importance of Gln H-bond is justified since ketones that do not form the interactions do not show any biofilm inhibitory activities against *C. albicans.*

Interestingly, the 2-undecanone molecule is a naturally occurring insect repellent currently marketed as IBI-246 [51]. This non-toxic molecule has been approved for use by US Environmental Protection Agency (EPA) against insects, especially ticks, mites, and mosquitoes [52]. The ketone was also non-toxic to a nematode model. Therefore, it is believed that 2-undecanone could be effectively repurposed as antibiofilm and anti-hyphal agents targeted against *C. albicans* infections. Additional studies are warranted to further elucidate the molecular mode of action of 2-undecanone.

## Data Availability

Not applicable.

## Supplementary Materials

The following supporting information can be downloaded at: https://www.mdpi.com/article/10.3390/pharmaceutics14050962/s1, Figure S1: Effect of AP and CSP on growth of bacterial pathogens; Figure S2: Effect of 2-nonanone and 2-heptanone on *C. albicans* biofilms; Figure S3: Growth curve of *C. albicans* in the presence and absence of 2-undecanone (0.02% and 0.05%); Figure S4: Effect of 2-undecanone on *C. albicans* growth in RPMI-1640 media; Figure S5: GCMS graph showing the peaks (a) of compounds (b) secreted by *L. plantarum* cells; Figure S6: Surface/volume view of interaction of 2-undecanone with hyphal wall protein 1 (Hwp1 protein); Table S1: Microbial strains used in this study.
