# Supplementary material for: The Bacillary Postbiotics, Including 2-Undecanone, Suppress the Virulence of Pathogenic Microorganisms
Source: Pharmaceutics. 2022 Apr 29;14(5):962. doi: 10.3390/pharmaceutics14050962 (PMC9143114; doi:10.3390/pharmaceutics14050962)
Supplement: Supplementary file 1 [file pharmaceutics-14-00962-s001.zip › pharmaceutics-1598081-supplementary.pdf]

pharmaceutics

The Bacillary Postbiotics Including 2-Undecanone Suppress the Virulence of Pathogenic Microorganisms

Satish Kumar Rajasekharan, and Moshe Shemesh *

Department of Food Sciences, Institute of Postharvest Technology and Food Sciences, Agricultural Research Organization (ARO), The Volcani Institute, Rishon LeZion 7528809, Israel

* **Corresponding author:** moshesh@agri.gov.il

Table S1. Microbial strains used in this study.

Strain	Specifications
L. planarum 3297	Isolate from healthy cow
C. albicans SC5314	Clinical specimen - human
E. coli OP50	Uracil auxotroph, Feed for *C. elegans*
E. coli 106878	Human pathogen
S. aureus 25923	MRSA

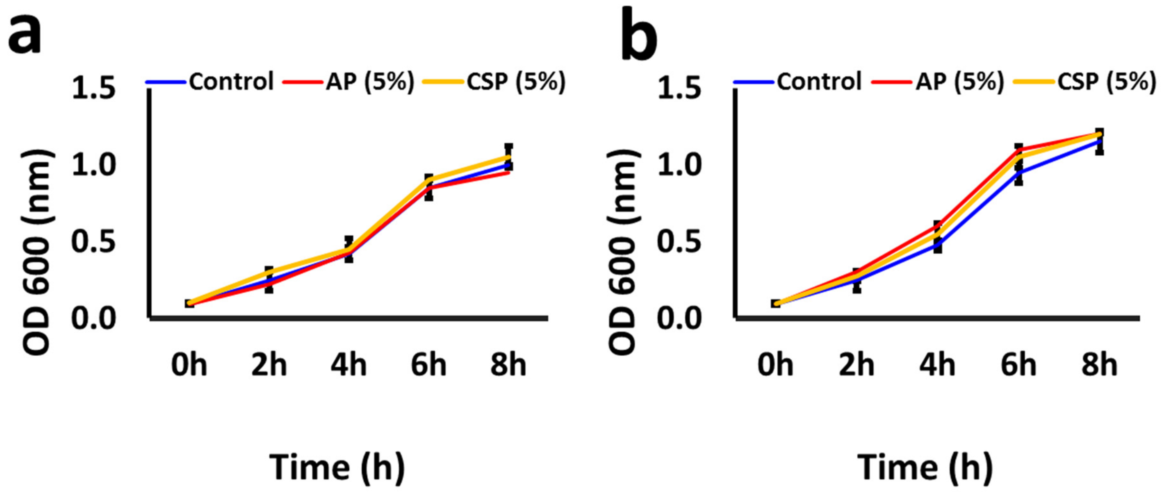

Figure S1. Effect of AP and CSP on growth of bacterial pathogens. (a) Effect of AP and CSP on *E. coli* growth. The graph shows the means ± SEMs of three measurements. * *P*>0.05 vs. the non-treated controls. (b) Effect of AP and CSP on *S. aureus* growth. The graph shows the means ± SEMs of three measurements. * *P*>0.05 vs. the non-treated controls.

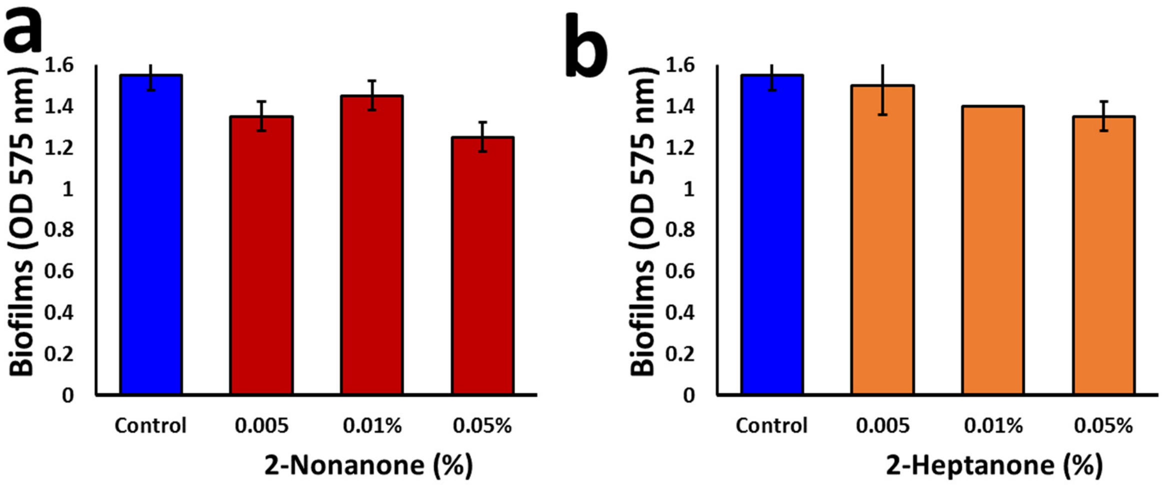

Figure S2. Effect of 2-nonanone and 2-heptanone on *C. albicans* biofilms. (a) Crystal violet quantifications of *C. albicans* biofilm formation on polystyrene surface in the presence and absence of 2-nonanone in PDB. The graph shows the means ± SEMs of three measurements. *P*>0.05 vs. the non-treated controls. (b) Crystal violet quantifications of *C. albicans* biofilm formation on polystyrene

surface in the presence and absence of 2-heptanone in PDB. The graph shows the means ± SEMs of three measurements. *P*>0.05 vs. the non-treated controls.

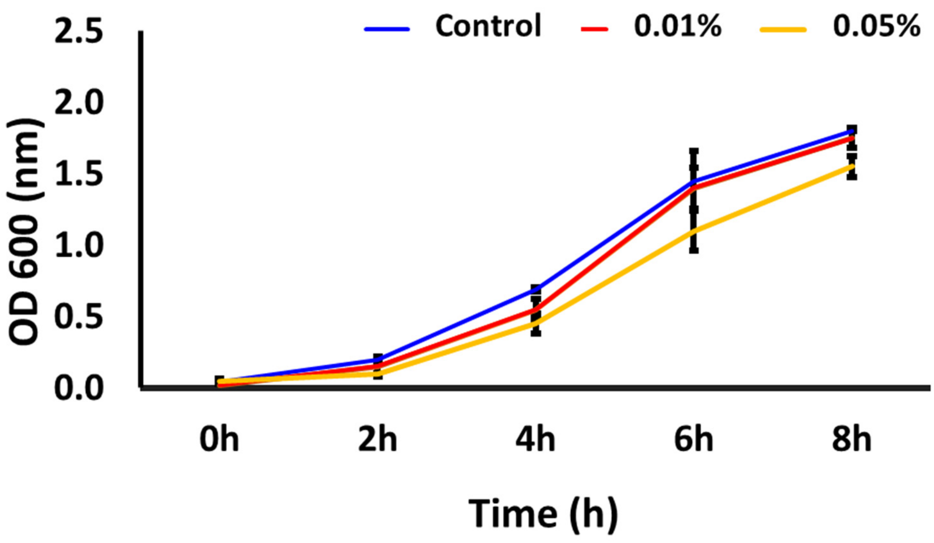

Figure S3. Growth curve of *C. albicans* in the presence and absence of 2-undecanone (0.02% and 0.05%).

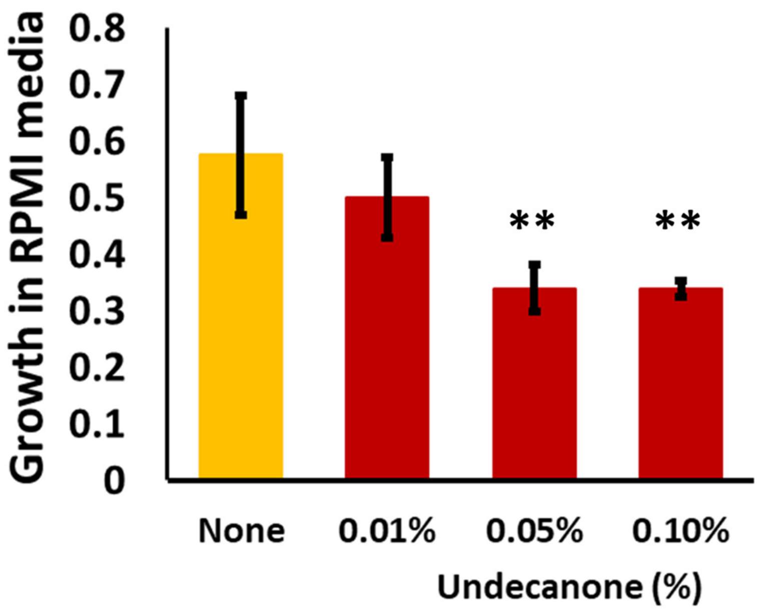

Figure S4. Effect of 2-undecanone on *C. albicans* growth in RPMI-1640 media. Crystal violet quantifications of *C. albicans* biofilm formation on polystyrene surface in the presence and absence of 2-heptanone in PDB. The graph shows the means ± SEMs of three measurements. *** *P*<0.001 and ** *P*<0.01 vs. the non-treated controls.

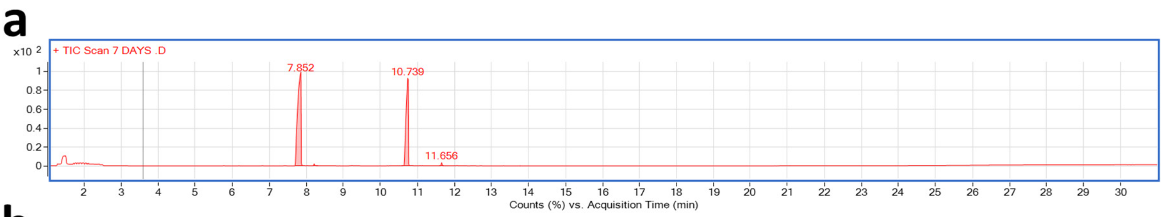

b

RT	Formula	Name	m/z	Mass (DB)	Score	Polarity	Height	Area
7.833	CH_3I	Methane, iodo-	142.2	141.9	69.26	Positive	2632132	27681087
7.846	$C_6H_{12}O$	Cyclopentanol, 3-methyl-	71.1	100.1	64.59	Positive	6607198	235930200
8.213	$C_9H_{18}O_2$	Octanoic acid, methyl ester	74.1	158.1	83.48	Positive	477538	2492202
10.743	$C_{11}H_{22}O$	2-Undecanone	58.1	170.2	76.09	Positive	8386575	221869995
11.654	$C_{13}H_{18}$	Naphthalene, 1,2,3,4-tetrahydro-1,1,6-trimethyl-	159.1	174.1	72.65	Positive	1058212	3651985

Figure S5. GCMS graph showing the peaks (a) of compounds (b) secreted by *L. plantarum* cells.

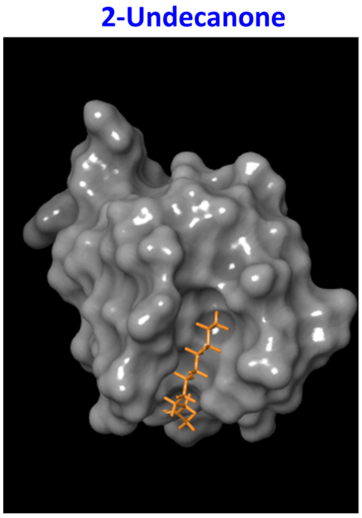

Figure S6. Surface/volume view of interaction of 2-undecanone with hyphal wall protein 1 (Hwp1 protein).
